# Unveiling the Power of Magnetic-Driven Regenerative Medicine: Bone Regeneration and Functional Reconstruction

**DOI:** 10.34133/research.0707

**Published:** 2025-05-22

**Authors:** Chenxi Xu, Pengzhen Cheng, Junxiang Wang, Beilei Zhang, Peng Shang, Yi Lv, Qiang Jie

**Affiliations:** ^1^ Pediatric Hospital, Honghui Hospital, Xi’an Jiaotong University, Xi’an, China.; ^2^ College of Life Sciences, Northwest University, Xi’an, China.; ^3^ Office of Medical Information Management, The First Affiliated Hospital of Xi’an Jiaotong University, Xi’an, China.; ^4^ Key Laboratory for Space Biosciences and Biotechnology, Northwestern Polytechnical University, Xi’an, China.; ^5^ Department of Hepatobiliary Surgery, The First Affiliated Hospital of Xi’an Jiaotong University, Xi’an, China.

## Abstract

To improve the treatment outcomes for large bone defects and osteoporosis, researchers have been committed to reducing bone loss and accelerating bone regeneration through cell transplantation, biomaterial intervention, and biophysical stimulation over the past few decades. Magnetism, as a noninvasive biophysical stimulus, has been employed in the repair of the musculoskeletal system, achieving a series of promising results. In this review, we provide a retrospective analysis and perspective of research on magnetic-driven bone regeneration and functional reconstruction. This review aims to delineate safe and efficient magnetic application modalities and to summarize the potential mechanisms by which magnetism regulates the behavior of skeletal lineage cells, thereby providing insights for the expansion and translational application of magnetic-driven regenerative medicine.

## Introduction

Biophysical stimuli, including mechanical forces, acoustic waves, light, thermal energy, electrical currents, and magnetic fields (MFs), represent an advanced strategy that is safe, efficient, and practical for tissue regeneration [1–3]. Magnetism, as one of the biophysical cues, possesses low toxicity and noninvasive characters. It is ubiquitous in nature and continuously influences human health. A multitude of studies have demonstrated the therapeutic potential of magnetism for a variety of diseases [4–9]. In particular, the synergy between magnetic-responsive materials and MFs has been proven to facilitate tissue regeneration and function reconstruction [10,11]. Magnetic-driven regenerative medicine (MDRM), based on magnetic-mediated regenerative strategies and cutting-edge technologies, has charted a viable path for functional organ regeneration.

In recent years, MDRM has also been increasingly applied in bone regeneration researches. The mechanical properties of magnetism are ideally suited to the mechanosensitive nature of skeletal lineage cells [12–20], rendering magnetic forces as one of the important pathways in magnetic-driven bone regeneration. Additionally, other forms such as MFs, magnetic hyperthermia, and magnetoelectric effects have been discovered to possess the potential to regulate the proliferation and differentiation of osteoprogenitor cells. This review endeavors to provide a systematic synthesis of the research advancements in the application of MDRM for bone regeneration over the past 35 years, with a pronounced focus on the progress achieved within the last decade. We delineate the concept of MDRM, the classification of magnetic materials and MFs, and their application approaches and mechanisms, with a particular emphasis on the role played by magnetic force. We also highlight the future development potentials and challenges of MDRM for bone regeneration.

## MFs and Magnetic Materials

### Magnetic fields

Recent findings indicate that MFs influence bone metabolism, prevent bone loss, and increase bone mass [21,22]. They act either in concert with magnetic responsive materials or as an independent therapeutic intervention. Table 1 summarizes the types of MFs, their parameters, disease models, and therapeutic effects in current research applying MFs without the use of magnetic materials. The predominant application modalities of MFs are divided into 2 categories: electromagnetic fields (EMFs) and static magnetic fields (SMFs). EMFs endow a fluctuating and oscillatory magnetic environment, whereas SMFs impart a stable and invariant magnetic influence. Although their modes of action differ, they both have the potential to accelerate osteogenic processes by activating various cellular signaling pathways and regulating the skeletal lineage cell functions.

**Table 1.** Both EMFs and SMFs have the potential to promote osteogenesis independently

Type of MF	Parameters	Cells/Animal model	Effects	Ref.
PEMF	0.01–0.04 T/s; 1 h/day	Ulnae in turkeys	Bone mass being controlled in the absence of mechanical loading	[23]
EMF	30/45 Hz; 1 mT	ADSCs	Osteogenic differentiation	[26]
PEMF	16 Hz; 45 day	HLS rat model	Controlling bone loss induced by simulated microgravity	[24]
PEMF	100 Hz; 1.5–1.8 G	Nonhealing bony defect in Wistar rats	Enhancing effect on the bone-inductive properties of the DBM	[25]
EMF	15 Hz, 1 mT, 4 h/day	BMSCs; critical-sized calvarial defect in rats	Enhancing proliferation and osteogenic differentiation capacity of BMSCs	[27]
PEMF	15 Hz; 2.4 mT; 2 h/day; 8 weeks	KK-Ay mouse	Resisting T2DM-associated bone deterioration	[29]
SMF	8 T	Mouse osteoblastic MC3T3-E1 cells	Stimulating bone formation	[33]
SMF	160 mT	Rat calvaria cell	Promoting osteoblastic differentiation and activation	[32]
SMF	0.2–0.6 T	BMSCs; bone mass loss induced by either Dex or ATRA in mice	Alleviating bone mass loss	[34]
SMF	130 mT	hABMSCs	Osteogenic potential of NHGH derived from hABMSCs	[35]

hABMSCs, human alveolar bone-derived mesenchymal stem cells; ATRA, all-trans retinoic acid; Dex, dexamethasone; HLS, hindlimb suspension; NHGH, natural human growth hormone

#### Electromagnetic fields

To prevent the osteoporosis that is concomitant with disuse, a study established an osteoporotic animal model and subsequently treated it with pulsed EMF (PEMF). Their findings indicated that short daily exposures to well-calibrated EMFs positively regulate the behavior of cellular populations crucial for bone remodeling. Moreover, they delineated an efficacious threshold of induced electrical power within which bone mass can be regulated devoid of mechanical loading [23]. Subsequent investigations have also elucidated the therapeutic potential of PEMFs in synergistic application. In a rodent model of simulated microgravity-induced osteoporosis, intraperitoneal administration of HAp combined with a PEMF synergistically attenuated bone loss [24]. Concurrently, another study demonstrated that the coordinated application of a PEMF with decellularized cancellous bone matrix (DBM) effectively promoted osteoblast differentiation [25]. These evidences collectively underscore the multimodal efficacy of PEMFs in bone regeneration strategies and the potential of relieving bone pain, preventing fractures, and avoiding a decline in mobility.

In addition, EMFs facilitate the osteogenic differentiation of MSCs, with the impact varying contingent upon the conditions of the applied EMFs [26,27]. The application of EMFs has also been shown to inhibit osteoclastogenesis [28], thereby improving bone microarchitecture and quality through regulating the biological functions of both osteoblast and osteocyte [29]. Consequently, the dose-dependent modulation of the aforementioned cells by EMFs demonstrates significant translational potential for augmenting osteogenic differentiation and promoting bone regeneration in compromised skeletal environments.

However, the generation of EMFs necessitates more sophisticated equipment, often tethered to a power source, which makes the development of wearable devices and the targeted stimulation of specific areas challenging. These factors impose certain constraints on the application of EMFs. Nonetheless, surmounting these challenges could unlock novel dimensions in scientific exploration and fully reveal the dynamic stimulation effects offered by EMFs. The application of organoid-on-chip technology now presents a novel opportunity for in-depth exploration of EMFs [30,31]. This advanced platform enables precise simulation of physiological microenvironments at a microscopic scale. Investigating the impact of EMFs on bone through this system could offer an efficient and controlled experimental approach, effectively minimizing confounding variables while maintaining high biological relevance.

#### Static magnetic fields

In contrast to the dynamic stimuli of EMFs, SMFs offer a uniform directionality and intensity of influence to cells and organisms constantly. The spectrum of SMF applications in magnetic-driven bone regeneration research spans from weak fields (<1 mT) up to high fields (>1 T). Collectively, these fields have been shown to stimulate bone formation by enhancing osteoblastic differentiation and activation [32]. Notably, high SMFs exhibit the dual capacity to stimulate bone formation and to regulate the alignment of osteoblasts [33]. Evidence has substantiated that moderate SMFs within the range of 0.2 to 0.6 T induce the osteoblastic differentiation of BMSCs in a manner that is dependent on the field’s intensity [34]. Furthermore, an SMF of 130 mT has been demonstrated to modulate the paracrine and autocrine signaling of growth factors, including vascular endothelial growth factor (VEGF) and bone morphogenetic protein-2 (BMP-2), as well as the secretion of natural human growth hormone by human alveolar bone-derived mesenchymal stem cells. Under the stimulation of the SMF, the cells showed an increase in metabolic activity, the expression of osteogenesis-related genes, and the formation of mineralized nodules [35].

Previous studies, including from our research group, have demonstrated that the equilibrium of bone homeostasis is the result of the osteoclast–osteoblast coupling [36]. Therefore, SMFs may regulate bone mass through exerting distinct effects on osteoclasts from those on osteoblasts. We have discovered that SMFs at intensities of 500 nT and 0.2 T promoted osteoclast differentiation, formation, and bone-resorptive activity, whereas a 16-T SMF exerted an inhibitory influence [37,38]. These effects elucidated that the SMF possess the potential to modulate bone mass and preventing the complications of osteoporosis, with the impact depending on the intensity of MF.

Furthermore, SMFs are often utilized to simulate conditions of microgravity or hyper gravity. Gravity is integral to the life cycle of organisms, shaping their growth and development. Our serial studies have revealed that under the simulated conditions of microgravity and hyper gravity induced by SMFs, there are apparent changes in the biological rhythms and the concentration of trace elements within organisms. Notably, the alteration of iron concentration could be a key mechanism through which SMFs may exert regulatory effects on osteoblasts and osteoclasts, thereby influencing the microstructure and mechanical integrity of bone [39–48]. These findings highlight the profound implications of SMFs on the intricate balance of bone homeostasis and the potential therapeutic applications in bone health and disease.

While EMFs and SMFs exhibit analogous effects in facilitating bone regeneration, their operational principles and practical implementations are markedly distinct. The apparatus for generating an SMF can be simplified to the level of a single magnet, underscoring its ease of use. Within the experimental environment, SMFs can be applied to the entire organism or, with effective magnetic shielding, to localized regions of interest. In the meantime, due to the absence of a requirement for alternating current, SMF generators often more readily produce high-intensity MFs compared to EMF devices. This utility affords it a more straightforward integration into scientific inquiry and clinical translation endeavors.

In clinical practice, PEMFs, which have proven benefits, have been approved by the Food and Drug Administration (FDA) for accelerating fracture healing, while SMF applications now face skepticism due to insufficient large-scale human trials and reliance on animal models. In the meantime, both SMFs and PEMFs encounter challenges in standardizing parameters, including optimal intensity, frequency, and exposure duration alongside long-term safety concerns, such as potential oxidative stress from prolonged exposure. Current limitations involve insufficient understanding of SMF’s cellular interactions and the lack of comprehensive clinical databases for personalized treatment protocols. In the future, researchers could focus on optimizing parameters, validating mechanisms, and conducting translational studies to establish SMFs as independent therapies, especially for osteoporosis and bone repair.

### Magnetic nanoparticles

**Fig. 1.**
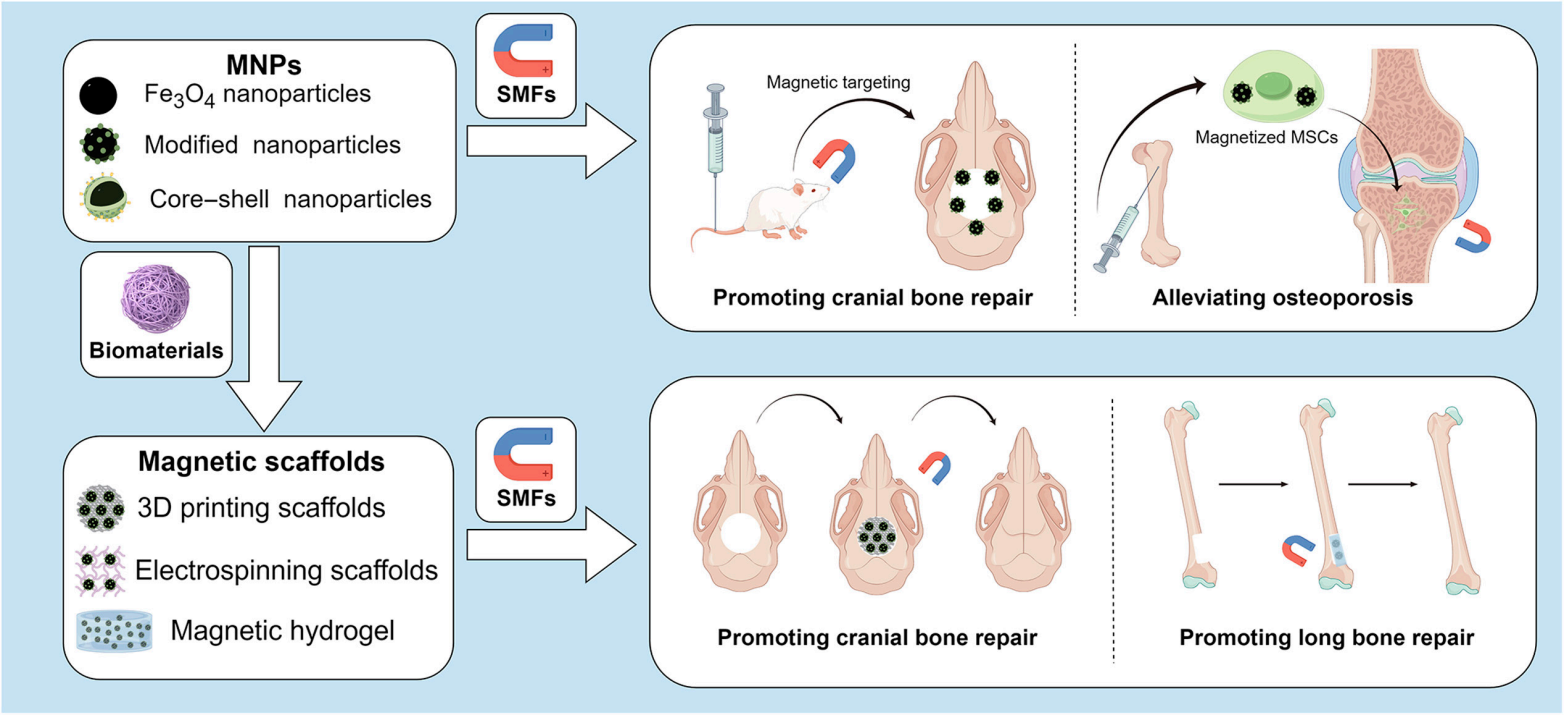
Magnetic materials (by Figdraw). MNPs include Fe_3_O_4_ nanoparticles, modified nanoparticles, and core–shell nanoparticles. They can be injected and promote bone repair or alleviate osteoporosis. Magnetic scaffolds can take the form of magnetic hydrogels or magnetic bioinks, fabricated through technologies such as 3D printing and electrospinning. They can be placed in situ and serve to facilitate bone repair under the influence of an MF.

Magnetic nanoparticles (MNPs) are one of the most commonly used magnetic materials, typically spherical in shape but can also be processed into nanorods, nanorings, core–shell structures, and other shapes according to their applications (Fig. 1). Superparamagnetic iron oxide nanoparticles (SPIONs), as a type of MNPs, have been widely applied in biomedical researches. SPIONs are primarily composed of Fe_3_O_4_ or γ-Fe_2_O_3_. They can be used directly [49] or modified in various ways to achieve different application goals. On the one hand, modifications with chemical macromolecules or other metal coatings, such as polyethylene glycol (PEG) [50], poly(lactic-co-glycolic acid) (PLGA) [51–53], amine hydrochloride/poly(styrene) sulfonate (PAH/PSS) [54], (3-aminopropyl) triethoxysilane (APTES) [55,56], polydopamine (PDA) [57], graphene oxide (GO) [58], SiO_2_ [59], polyacrylic acid (PAA) [60], meso-2,3-dimercaptosuccinic acid (DMSA) [61,62], and gold [63], can stabilize the structure of MNPs and enhance their biocompatibility. On the other hand, biomolecules, including microRNAs [64,65], peptides [62,66–69], proteins [70], antibodies [71–74], and drugs [75], endow MNPs with a richer biological functionality, making them more efficient in targeted cell binding and promotion of cellular gene expression. These molecules may also enhance cellular magnetization efficiency [62,76], scavenge reactive oxygen species (ROS) [58], induce differentiation of stem cells, and label cells for further tracing [61,77–80]. In summary, MNPs can not only exert their influence through magnetic responsiveness, but also extend their biological functions with the help of loaded biomolecules. This dual capability allows for a range of applications, from diagnostic imaging to therapeutic interventions. Table 2 briefly lists the types of MNPs, their particle size, functions, and the cell and animal models. Their specific applications will be described in detail in the section on magnetic effects.

**Table 2.** MNPs for bone regeneration

Characteristics	Diameter	Function	Cell type	Animal model	Ref.
Fe_3_O_4_ MNPs	6.6 nm	Controlling ADSCs fate by magnetic mechanical stimulation	ADSCs	-	[49]
Magnetic aggregation-induced bone-targeting PLGA-based nanocarrier	200–300 nm	Magnetic aggregation-induced property	BMSCs; HUVECs	OVX-induced osteoporosis mouse model	[51]
Magnetic PLGA microspheres loaded with SPIONs	160–200 μm	Promoting the reconstruction of bone defects through regulating the BMSCs under an MF	BMSCs	Femur bone defect rat model	[52]
Magnetic microcapsules with PAH/PSS shell and FITC-BSA label	30 nm	MSCs with internalized magnetic capsules respond to an MF	MSCs	-	[54]
Fe_3_O_4_ MNPs modified with APTES and rhodamine B	-	Creating magnetic cell sheets	MC3T3-E1; hADSCs	-	[55]
Fe_3_O_4_ MNPs modified with APTES and rhodamine B	5–15 nm	Construction of stratified magnetic responsive heterotypic cell sheets; osteogenesis without osteogenic differentiating factors	HUVECs; ADSCs	CAM model	[56]
Fe_3_O_4_ MNPs modified with polydopamine	55–60 nm	Magnetic targeting; inhibiting the apoptosis of osteoblasts and enhancing osteogenic effect under an MF	hUCMSCs	ONFH rat model	[57]
GO-modified Fe_3_O_4_ MNPs	12 nm	H_2_O_2_ and hydroxyl radical scavenging; intracellular ROS scavenging; loading BMP-2 and inducing osteogenic differentiation of MSCs under an SMF	MSCs	-	[58]
MSNPs (SiO_2_-coated MNP)	29.07 nm	Spatial localization, cell retention, and directional tracking capabilities under an MF; promoting osteogenesis, mineralization, and angiogenesis under an MF	MSCs	OVX-induced osteoporosis mouse model	[59]
DMSA-coated γ-Fe_2_O_3_ nanoparticles	5–18 nm	Good biocompatibility; as tracers for MSCs	MSCs	-	[61]
DMSA-coated SPIONs with RGD peptide	94.2 ± 6.4 nm	Targeting integrin receptors; promoting osteogenic differentiation of BMSCs under an MF	hBMSCs	-	[62]
Gold-coated MNPs	-	Loading extracellular vesicles and promoting the osteogenesis of DO rats	mBMSCs; mouse osteoblasts	DO rats model	[63]
nHAp-based composite co-doped with MNPs functionalized with microRNAs	nHAp: 25–56 nm × 4–16 nm	Improving metabolism of pre-osteoblasts and osteogenesis; decreasing differentiation of pre-osteoclasts; releasing therapeutic miRNAs under an MF	MC3T3-E1; 4B12; RAW 264.7	-	[64]
Mesoporous silica and Fe_3_O_4_ composite-targeted nanoparticles loaded with baicalein	100 nm	Magnetically delivering; inducing macrophage recruitment and polarizing them toward the M2 phenotype; inducing MSCs toward osteoblastic differentiation	Mouse BMDMs; mMSCs	Murine fracture model	[66]
SPIO carboxyl functionalized MNP were covalently coated with UM206 peptide	250 nm	Activating Wnt signaling	hMSCs	Chick fetal femur culture and microinjection	[68]
Hybrid microspheres made by recombinant proteins and iron-doped HAp	50–75 μm	Loading rhBMP-2 and releasing drug under PEMFs	hMSCs	-	[69]
SPIONs-loaded BSA	140–190 nm	Enhancing the osteogenic differentiation of MSCs under an SMF	MSCs	-	[70]
Anti-human PDGFRα antibody-conjugated SPIONs and RGD-conjugated SPIONs	250 nm	Affecting osteogenesis of hMSCs significantly by mechanical stimulation via magnetic tagging	hMSCs	-	[72]
RGD and TREK-1 antibody functionalized carboxyl-coated SPIONs	300 nm	Delivering mechanical stimuli by directly targeting cell-surface mechanosensors and transducing forces from an MF	hBMSCs	Chick fetal femur culture and microinjection	[71,73]
TREK-1 antibody functionalized carboxyl-coated MNPs	250 nm	Remotely delivering mechanical stimuli to the mechano-receptor	MSCs	Preclinical ovine model of bone injury	[74]
SPIONs-coated hairbot	10 μm of thickness and 60–80 μm of lateral dimension	As ultrasonography contrast agent; promoting osteogenesis under magnetic actuation; drug delivery; good biocompatibility	mMSCs	Swiss albino mouse	[75]
SPIONs	-	Chondrocyte labeling	Rabbit chondrocytes	-	[77]

RGD, Arg-Gly-Asp; nHAp, nanohydroxyapatite; MSCs, mesenchymal stem cells; hMSCs, human mesenchymal stem cells; mMSCs, mouse mesenchymal stem cells; BMSCs, bone marrow mesenchymal stem cells; hBMSCs, human bone marrow mesenchymal stem cells; mBMSCs, mouse bone marrow mesenchymal stem cells; hUCMSCs, human umbilical cord mesenchymal stem cells; BMDMs, bone marrow-derived macrophages; ADSCs, adipose-derived stromal cells; hADSCs, human adipose-derived stromal cells; HUVECs, human umbilical vein endothelial cells; DO, diabetic osteoporosis; MSNPs, magnetic silica nanoparticles; BSA, bovine serum albumin; ONFH, osteonecrosis of the femoral head; OVX, ovariectomy; PEMF, pulsed electromagnetic field; CAM, chick chorioallantoic membrane;

### Magnetic scaffolds

Magnetic scaffolds, another kind of magnetic materials, are predominantly fabricated by integrating biomaterials with MNPs. These biomaterials can be of natural origin, synthetic, or a hybrid of both [81–83]. The design of magnetic scaffolds ranges from macroscopic structures to microscopic entities like microcarriers or microrobots [84–86]. The fabrication techniques for these scaffolds are diverse. Some studies employ straightforward mixing and stirring of raw materials [60], while others utilize advanced methods such as electrospinning [87–89] and 3D printing [90] to produce scaffolds that meet specific research criteria (Fig. 1). It is well recognized that the pore size, mechanical properties, and micromorphology of biomaterials determine their in vitro and in vivo application outcomes [91–93]. For magnetic scaffolds, which are encapsulated biological factors, the surface modification of embedded MNPs and their responsiveness to external MFs are equally pivotal [94–101]. The incorporation of MNPs not only directly alters the characteristics of biomaterials, but also endows them with dynamic changes in mechanical properties and micromorphology upon exposure to an external MF. This magnetic responsive behavior is a distinctive feature of magnetic scaffolds, setting them apart from other grafts. Table 3 summarizes the raw biomaterials, fabrication approaches, functional characteristics of the magnetic scaffolds, and the types of cells they support and the animal models they are integrated into, as applied in magnetic-driven bone regeneration.

**Table 3.** Magnetic scaffolds, which can take any forms, facilitate bone formation under the influence of an external MF

Raw biomaterials	Preparation method	Function	Loaded cells in in vivo experiments	Animal model	Ref.
Fe_3_O_4_ nanoparticles; chitosan/PEG hydrogel	Mixing	Enhancing osteogenic differentiation of the MSCs by increasing temperature under an EMF	-	-	[50]
Silk fibroin hydrogel-loaded PAA modified Fe_3_O_4_ nanoparticles	Mechanical stirring	H_2_O_2_ and hydroxyl radical scavenging; intracellular ROS scavenging; promoting osteogenic differentiation of MSCs under an SMF	-	-	[60]
Porous HAp scaffold; SPIONs-loaded VEGF	Mixing	Releasing VEGF under magnetic stimulation; stimulating bone formation; improving bone/scaffold interaction	-	Sheep metatarsus critical size defect model	[67]
DBM-incorporated MNPs	Molding in Teflon holders	Promoting bone regeneration and angiogenesis with a low-frequency PEMF	-	Rat bilateral critical-size cranial defect model	[78]
Type I collagen; PEG-modified MNPs	Genipin-crosslink	Enabling efficient polarization of encapsulated macrophage to the M2 phenotype in response to SMF	Macrophage	Rat critical-sized calvarial defect model	[85]
Glycosylated SPIONs-loaded BMP-2; agarose hydrogel	Mixing	Spatially directing the osteogenesis of hMSCs	-	-	[86]
Magnetic γ-Fe_2_O_3_ nanoparticles with a coating of DMSA; nHAp; poly lactide; DMAc	Electrospinning	Macrophage phenotypic mechano-modulation under an SMF	-	Mouse subcutaneous implantation in model	[87]
γ-Fe_2_O_3_ nanoparticles coated with DMSA; nHAp; DMAc; PLA	Electrospinning	Promoting the wound-healing phenotype of fibroblasts under an SMF; accelerating the osteogenesis of pre-osteoblasts under an SMF	-	-	[88]
PVP or citric acid-coated MNPs; gelatin; HFIP	Electrospinning	Enhancing chondrogenesis and osteogenesis by mechanical stimulation under an EMF	Rabbit BMSCs	Rabbit osteochondral defects	[94]
PBLG-g-IONPs; PLGA	Mechanical stirring	Being beneficial for the adhesion and osteogenic differentiation of pre-osteoblasts under an SMF	-	-	[95]
Dicalcium phosphate anhydrous; γ-Fe_2_O_3_ nanoparticles	Mechanical stirring	Enhancing the osteoinduction of hDPSCs under an SMF	-	Rat critical-sized buccal bone defect model	[110]
Oleic acid modified SPIONs; PLGA	Mechanical agitation	Improving cell attachment and osteogenic differentiation under an SMF	-	-	[113]
Type I collagen; NH_4_H_2_PO_4_; Ca (NO_3_)_2_; PEG-modified MNPs	Electrodeposition	Regulating the osteogenic potential of BMSCs through mechanical stimulation under different magnetic actuation directions	-	Rat cranial defect model	[106]
Type I collagen; NH_4_H_2_PO_4_; Ca (NO_3_)_2_; PEG-modified MNPs	Mixing and AP-ECD	Improving macrophage M2 polarization and thereby facilitating osteogenesis using an external MF;	-	Rat cranial defect model	[114]
Type I collagen; NH_4_H_2_PO_4_; Ca (NO_3_)_2_; PEG-modified MNPs	Mixing and AP-ECD	Magnetically actuated mechanical stimuli enhancing osteogenic differentiation of the MC3T3-E1 cells under an SMF	-	-	[112]
Type I collagen; NH_4_H_2_PO_4_; Ca (NO_3_)_2_; PEG-modified MNPs	Mixing and AP-ECD	Magnetically actuated mechanical stimuli enhancing osteogenic differentiation of the MSCs under a periodic SMF	-	-	[107]
CI particles; polyacrylamide hydrogels	Mixing	Magnetic mechanical stimuli promoting proangiogenic molecule secretion and osteogenesis dynamic control of MSCs	-	-	[108]
MNP; GelMA	Electrospinning and mixing	Reversibly stiffening the material by SMF, thereby mechanical stimulating encapsulated cells	-	-	[111]
Nanoporous alumina templates; Fe nanowire	Electrodeposition	Magneto-mechanically modulated nanosurface enhancing the osteogenic differentiation capabilities of BMSCs	-	-	[217]
Silk fibroin; IONPs	Stirring	Promoting a faster and better osteogenic differentiation of hMSCs, particularly under an MF	-	-	[109]
GelMA; PVA; PTH; MNPs	Mixing; photocuring	Providing magnetic mechanical stimulation to enhance osteogenic commitment; as a PTH reservoir for programmed release	-	Rat critical-sized calvarial defect model	[116]
MgO; Fe_3_O_4_ nanoparticles; PLA	Selective laser sintering	Using SMF to regulate the activity of MAGT1 on the membrane of rat BMSCs	-	Rat skull defect model	[118]
Macroporous gelatin sponges; ferrogel	Mixing	Delivering SDF-1α and BMP-2; regulating and optimizing the osteodifferentiation process of mMSC	-	-	[115]
Methacrylated chondroitin sulfate; platelet lysate; MNPs	Mixing	Manipulating EMF and making the magnetic hydrogel releasing growth factors	-	-	[117]
SPIONs; PLGA-COOH/HAp	Mixing	Antitumor and osteogenic capacity	-	Rabbit’s ulna defect model	[132]
Mg macroscale rods	-	Enhancing the maturation of DCs and the mild magnetic hyperthermia therapy induced the immunogenic cell death in osteosarcoma cells	-	BalB/c mouse femoral bone osteosarcoma	[139]

DCs, dendritic cells; hDPSCs, human dental pulp stem cells; VEGF, vascular endothelial growth factor; DBM, decellularized cancellous bone matrix; DMAc, *N*,*N*-dimethyl acetamide; PLA, poly(lactic acid); PBLG-g-IONPs, poly(γ-benzyl-l-glutamate)-grafted iron oxide nanoparticles; NH_4_H_2_PO_4_, ammonium dihydrogen phosphate; Ca (NO_3_)_2_, lime nitrate; AP-ECD, alternating potential-assisted electrochemical deposition; CI, carbonyl iron; GelMA, gelatin methacryloyl; PVA, polyvinyl alcohol; PVP, polyvinylpyrrolidone; PTH, parathyroid hormone; HFIP, 1,1,1,3,3,3- hexafluoro-2-propanol; hTDCs, human tendon-derived cells

Current research on MNPs and magnetic scaffolds has established a robust foundation in MDRM. Researchers can readily design and synthesize novel magnetic materials centered on MNPs for domain-specific applications. However, the use of MNPs as drug delivery vehicles represents merely one facet of their multifunctional utility. The diverse magnetic effects generated by MNPs and magnetic scaffolds under applied MFs warrant more scientific attention. These effects have already demonstrated remarkable efficacy in magnetic-driven bone regeneration.

## Magnetic Effects in Bone Regeneration

**Fig. 2.**
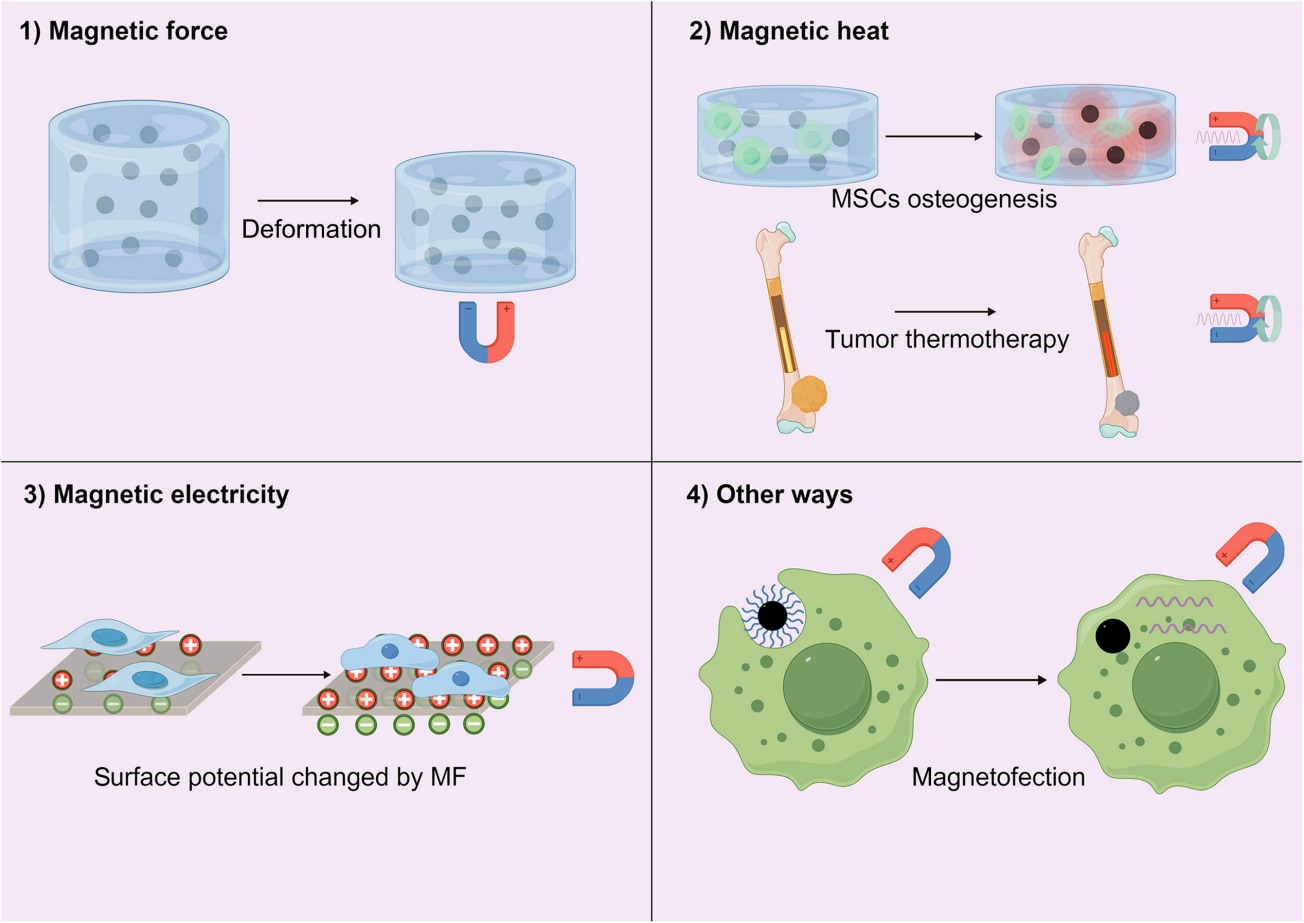
Magnetic effects in bone regeneration (by Figdraw). (1) Magnetic hydrogels exhibit deformation in response to MFs. (2) Magnetic hydrogels heat up under the influence of an EMF, facilitating the osteogenic differentiation of encapsulated MSCs. Eddy thermal effect of biodegradable magnesium rod, when subjected to an EMF, showed outstanding cytotoxic effects. (3) Changes in the membrane potential in response to an external MF stimulate the osteogenic differentiation of cells on its surface. (4) miRNA-carrying MNPs are internalized by cells under the influence of an MF, where they subsequently degrade to release RNA, thereby modulating cellular expression.

Under the influence of an external MF, MNPs and magnetic scaffolds accelerate bone regeneration through composite biophysical stimulation including mechanical load, thermal effects, and electrical stimulation (Fig. 2). The diverse magnetic effects significantly broaden the application strategies and scenarios for MDRM, making rapid bone regeneration and functional reconstruction a tangible possibility.

### Magnetic force

**Fig. 3.**
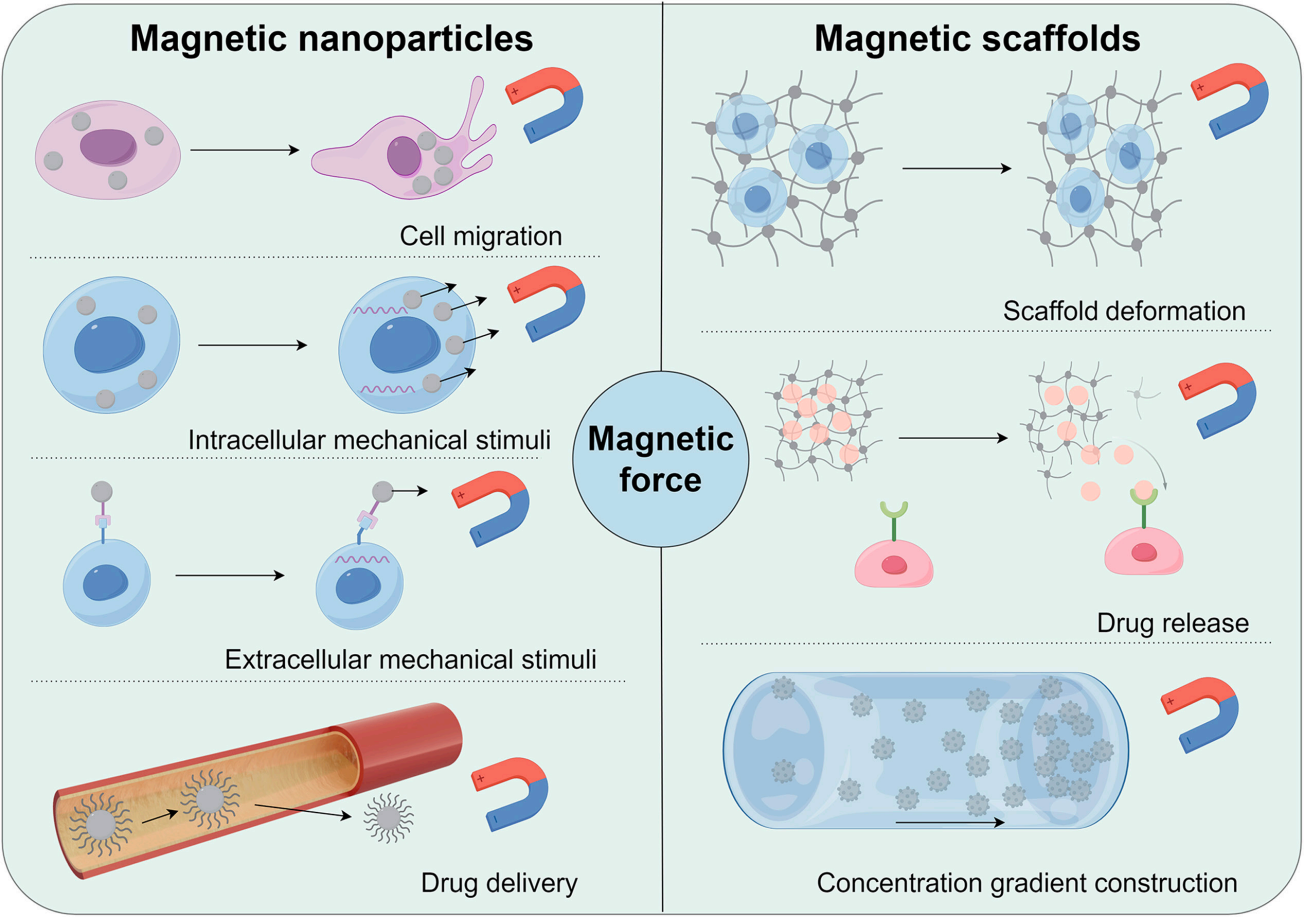
Application ways of magnetic force (by Figdraw). MNPs can be utilized for guiding cell migration, applying intracellular and extracellular mechanical stimuli to cells and targeted drug delivery. Magnetic scaffolds, on the other hand, impose extracellular mechanical stimuli through deformation and can also be employed for drug release and the construction of drug concentration gradients to elicit biochemical stimuli in cells.

The notion that mechanical load regulates bone regeneration has gained widespread acceptance [14]. Our recent study highlighted the critical regulatory effect of micromechanics on the chondrogenic differentiation of BMSCs and the subsequent process of endochondral ossification [102]. Interestingly, the mechanical effects generated by MFs, including micromechanics, also provide controllable mechanical stimulation to cells via magnetic materials. Figure 3 illustrates the application schemes of 7 distinct magnetic force effects utilized to achieve corresponding scientific goals. These MNPs and magnetic scaffolds are engineered to interact with MFs, harnessing mechanical forces to execute their biological functions.

After endocytosis, MNPs regulate cellular behavior by inducing mechanical stimuli within the magnetized cells under an external MF. Current findings have demonstrated that the osteogenic differentiation potential of magnetized MSCs and ADSCs is markedly augmented in the presence of an MF [49,70]. Meanwhile, recognizing the indispensable role of angiogenesis in bone repair [103,104], some researchers have advanced the concept of utilizing MFs to assemble magnetized human umbilical vein endothelial cells (HUVECs) and ADSCs into magnetic responsive cell sheets. This innovative approach is proved to enhance osteogenic outcomes [56]. Furthermore, the directed movement and enrichment of magnetized cells under the guidance of MFs offer a highly predictable approach for targeted therapeutic interventions within the body [52,54,55,57,59]. In an in vivo research lead by Jiang, magnetized MSCs were injected into ONFH (osteonecrosis of the femoral head) rats through tail vein and eventually not only promoted bone repair of the femoral head but also enhanced the function reconstruction under the guidance of an external MF [57].

The extrinsic application of mechanical stimuli via MNPs represents another approach for osteogenic induction. El Haj et al. have been at the forefront of this field. They have harnessed the MNPs with surface modifications to selectively engage with cell membrane receptors or mechanosensors such as the mechanically gated TREK1 K^+^ channel [71,73], RGD-binding domains [73], and Frizzled [68]. By subjecting these targeted cells to magnetic mechanical stimulation through an external MF, they have successfully elevated the mineral-to-matrix ratio, indicative of enhanced osteogenic activity. Their in vivo studies have further revealed that such targeted stimulation of exogenous stem cells not only promoted the osteogenic differentiation of the seed cells but also triggered the release of bioactive factors. These factors, in turn, improved collagen deposition and mineralization within the local cellular microenvironment [72].

Moreover, using external MFs for the targeted delivery of drugs represents an innovative and effective strategy for MDRM. Researchers have successfully applied MNPs as vectors for a range of therapeutic agents, such as doxorubicin [75], baicalein [66], microRNA [63,64], recombinant human bone morphogenetic protein-2 (rhBMP-2) [69], and the Piezo1-activatable molecule Yoda1 [51]. MNPs can also combine with exosomes to build a multipotent exosome platform for more accurate navigation and targeted delivery [63,105]. By utilizing the magnetic guidance system, these MNPs have been successfully targeted to bone defect sites, where they have been shown to stimulate osteogenic differentiation and enhance the reparative processes within the osseous microenvironment.

The utilization of magnetic scaffolds presents a more straightforward approach compared to the use of MNPs. Based on the responsiveness of MNPs to external MFs, biomaterials loaded with MNPs experience force-induced deformation when subjected to an external MF. This modification in mechanical properties exert stimulatory effects on the encapsulated cells. A multitude of studies have illustrated that the variations in the stiffness and the direction of deformation of magnetic scaffolds within an MF impact a diverse range of cell types. They are capable of not only regulating the osteogenic differentiation of MSCs [106–109], human dental pulp stem cells (hDPSCs) [110], ADSCs [111], MC3T3-E1 cells [112], and osteoblasts [113], but also indirectly facilitating bone reconstruction by modulating the phenotypes of macrophages [85,87,114] and fibroblasts [88], thereby coordinating a complex cellular response that drives in bone regeneration.

Beyond the mechanical stimulation they provide, magnetic scaffolds are frequently engineered to incorporate a suite of biochemical factors, thereby acquiring a multifaceted functionality. These scaffolds, under the manipulation of an external MF, are capable of delivering a controlled release of various osteoinductive agents, such as recruitment factors named stromal cell-derived factor 1α (SDF-1α) [115], BMP-2 [115], parathyroid hormone (PTH) [116], platelet lysate (PL) [117], and Mg^2+^ [118]. This programmed release is designed to attract endogenous stem cells to the site of injury, enhancing the osteogenesis process and refining therapeutic outcomes for bone repair.

Besides straightforward mixing, the integration of biochemical factors into magnetic scaffolds can be achieved through modifying MNPs embedded within the scaffold matrix. Under the guidance of an external MF, these MNPs, loaded with bioactive molecules, generate a concentration gradient through magnetic force-directed assembly [67,86]. This approach facilitates a sophisticated, multidimensional strategy for the repair and regeneration of both bone and cartilage tissues, enhancing the precision and efficacy of tissue engineering interventions.

### Magnetic heat

It is well known that the thermal effect of magnetic materials in EMFs has been a key method in immunoregulation and antitumor research [119–123]. Moreover, magnetic hyperthermia has become a significant application strategy for MDRM in bone regeneration. Magnetic hyperthermia is predominantly utilized for the treatment of osteosarcoma and the reconstruction of bone defects following bone tumor excision. Magnetic bioactive glass nanoparticles, as one of the magnetic materials applied in magnetic hyperthermia, are capable of heating to over 40 °C under an external MF, thereby achieving the ablation and inhibition of tumor cells [124–131]. Furthermore, these particles are often engineered with a mesoporous structure to control the sustained release of antineoplastic drugs, aiming to accomplish synergistic therapeutic effects [124–128]. Other magnetic materials, including magnetized HAp and Mg_2_SiO_4_-CoFe_2_O_4_, also possess similar therapeutic effects [132–137]. In another study, a triple-functional magnetic gel (Fe_3_O_4_/GOx/MgCO_3_@PLGA) with liquid–solid phase transition capability had been developed as a magnetic bone repair hydrogel (MBR). It drove magnetic thermal effects to simultaneously trigger GOx release, inhibit ATP production, and reduce HSP expression. By in situ injection, this magnetic hydrogel effectively suppressed tumor growth in osteosarcoma tumor-bearing mice and accelerated the reconstruction of bone defects through enhanced osteogenic differentiation and magnesium ion release [138]. In addition to in situ filling of nanoparticles or scaffolds, researchers also used biodegradable magnesium macroscale rods, acting as eddy thermo-magnetic agents under a low external EMF, combined with immunotherapy to treat osteosarcoma. The thermo effect of magnesium rods showed outstanding cytotoxic effects and enhanced the maturation of dendritic cells, and the mild magnetic hyperthermia therapy induced immunogenic cell death in osteosarcoma cells. Additionally, the biodegradable magnesium rods promoted bone osteogenesis [139].

Furthermore, the therapeutic applications of magnetic hyperthermia have been expanded to the repair of infected bone defects, emerging as a significant focus of contemporary research. In a latest study, a borosilicate bioactive glass and MNPs scaffold was investigated for implant-related *Staphylococcus aureus* bone infection. The scaffold demonstrated not only favorable immunomodulatory and antibacterial properties but also facilitated the formation of new bone at the original infection site [140]. A similar therapeutic effect was reported in another study in which MNPs had been integrated into magnesium calcium phosphate bone cement, with the addition of gelatin to refine the material. This composite undergoes controlled degradation, creating an alkaline microenvironment that not only neutralizes bacterial activity but also promotes the osteogenic differentiation and mineralization processes in BMSCs [141]. Yang et al. [142] recently used the eddy-thermal effect of magnesium (Mg) implants under an EMF for the controlled release of H_2_ gas and ions (OH^-^ and Mg^2+^) for the treatment of osteomyelitis. H_2_ and OH^-^ disrupted the environment of bacterial infections and impaired the permeability of bacterial membranes, while Mg^2+^ accelerated the process of bone repair.

However, limited research has been dedicated exclusively to the application of magnetic heat for the promotion of bone regeneration. Although hydrogels embedded with MNPs have been shown to well-induct osteogenic differentiation in MSCs when exposed to EMFs, even surpassing the effects of direct thermal stimulation [50], it remains to be elucidated whether the bone regenerative effects are a consequence of the MF or the heat generated by the MNPs. Obviously, heat energy beyond the limit of body temperature that can ablate tumor cells will inevitably also have adverse effects on normal cells. As the factors that promote bone regeneration in the current study are diverse, the direct role of thermal energy in magnetothermal induced bone regeneration is still debated. On the one hand, elements such as calcium, phosphorus, silicon, iron, zinc, and magnesium consisted in bioactive glass, HAp, and other metal-containing magnetic materials are inherently beneficial for bone tissue reconstruction [104,143–147]. On the other hand, the characteristics of injectable materials endow them with advantages that facilitate bone integration at the biomaterial interface.

In conclusion, these approaches exemplify the strengths of MDRM in addressing complex pathological conditions, but the therapeutic mechanism of magnetic thermal effects in promoting bone repair remains to be further explored. The application of an ideal treatment strategy should involve stringent selection of magnetic materials and precise control of parameters to achieve both tumor suppression and bone repair without damaging healthy tissues.

### Magnetic electricity

The intrinsic relation between magnetism and electricity has inspired scientific inquiry into the use of electromagnetic stimulation as a noninvasive, noncontact method to enhance bone defect healing. Current investigative efforts are directed toward the development of piezoelectric materials that respond to magnetic stimuli. The operational mechanism involves subjecting magnetostrictive MNPs within a polymer matrix to an external MF, causing them to deform and thereby induce a change in the polymer’s conformation and a concomitant alteration in surface charge due to piezoelectricity [148]. Materials such as poly(vinylidene fluoride) and its copolymers with terpolymers, enriched with cobalt ferrite (CFO), terbium-dysprosium iron alloys, or core–shell nanoparticles like CoFe_2_O_4_@BaTiO_3_, are adept at dynamically modulating the osteogenic differentiation of BMSCs by adjusting the surface potential in response to MFs [149–152]. Furthermore, EMFs generate inductive electric fields, suggesting that even without magnetoelectric materials, stimulation of EMFs has the potential to act on biological tissues through electrical stimulation. Noninvasive endogenous electrical stimulation represents another innovative technology of MDRM in bone repair strategies. However, research focusing on the osteogenic effects of EMFs through electrical stimulation is still in its infancy.

### Other ways

Beyond the previously discussed effects, MDRM also contains the innovative application of MNPs for the intracellular delivery of genetic materials under the influence of EMFs, a process known as magnetofection. These nanoparticles, once internalized by target cells, facilitate the release of nucleic acids or plasmids, thereby intervening in the gene expression machinery and directing cellular differentiation [65,153–155]. Furthermore, MNPs can be integrated with nanosensors to provide a means of detecting gene expression within cells [156]. This novel transfection approach has significantly improved the efficiency when compared to conventional transfection techniques [155]. The potential for controlling the release of genetic materials post-magnetic transfection gives the possibility for the precise spatiotemporal manipulation of cellular functions, enabling a new era of controlled gene therapy in MDRM.

The studies above highlighted the direct involvement of magnetic materials and MFs in the processes of bone regeneration and function reconstruction. In the meantime, exosomes derived from magnetic induction in vitro can also be used for bone regeneration. For instance, by co-culturing BMSCs with MNPs under an SMF, researchers have successfully isolated exosomes enriched with miR-1260a. These exosomes exert a pivotal influence on osteogenic and angiogenic activities [157]. Notably, their therapeutic application bypasses the direct stimulation of organisms by magnetic materials and MFs, enhancing the feasibility and safety of such treatments in a clinical context, marking a further extension of MDRM.

Therapeutic strategies leveraging magnetic effects remain in a nascent stage of development. Despite numerous innovative conceptualizations, their practical implementation faces formidable challenges. For instance, the current lack of sophisticated systems for precise spatiotemporal control of magnetic materials, coupled with the technical difficulty of achieving localized magnetic stimulation in biological systems under noninvasive conditions, represents significant bottlenecks. Addressing these challenges will likely necessitate interdisciplinary collaboration spanning biology, physics, and materials science to engineer advanced MDRM platforms. Furthermore, future research could expand the functional repertoire of MNPs by integrating complementary responsiveness to light, pH, and acoustic [158] stimuli. Such multimodal synergistic therapeutic approaches may prove instrumental in addressing complex orthopedic pathologies characterized by heterogeneous tissue microenvironments.

### Mechanisms of magnetic effects

Significant advancements have been made in the application of MDRM for bone regeneration. Concurrently, the potential mechanisms underlying these effects are being elucidated through the progression of detection technologies. An early study suggested that the osteo-inductive effects of PEMFs were, at least in part, mediated by enhancing the expression of *Bmp2* and *Bmp4* mRNA [159]. Now, some researchers have developed magnetic materials capable of activating Smad and Wnt signaling pathways of MSCs, thereby enhancing their potential for attachment, proliferation, and osteogenic differentiation [160–162]. Other studies focused on the interactions between the protein corona on magnetic HAp scaffolds and the immune system, or reveal the role of magnetic HAp scaffolds in promoting bone regeneration by enhancing endoplasmic reticulum stress and promoting osteoclast apoptosis [163,164]. In addition, the mechanisms of MFs themselves, magnetic forces, and the role of iron released from iron-containing magnetic materials are all of interest to scholars.

### Effects of MFs on cells

Although MFs were broadly categorized into 2 types in the preceding text, their application strategies in practice are quite diverse. EMFs exhibit a variety of waveforms, such as sinusoidal or square, each with distinct biological implications. Meanwhile, the implementation of SMFs extends beyond the static presence of a permanent magnet. Utilizing devices that generate relative motion between the SMF and biological entities enables the emulation of dynamic MF conditions. This dynamic simulation holds the potential to enrich our understanding of how MFs influence biological processes and to expand the therapeutic repertoire of magnetic-based interventions.

**Fig. 4.**
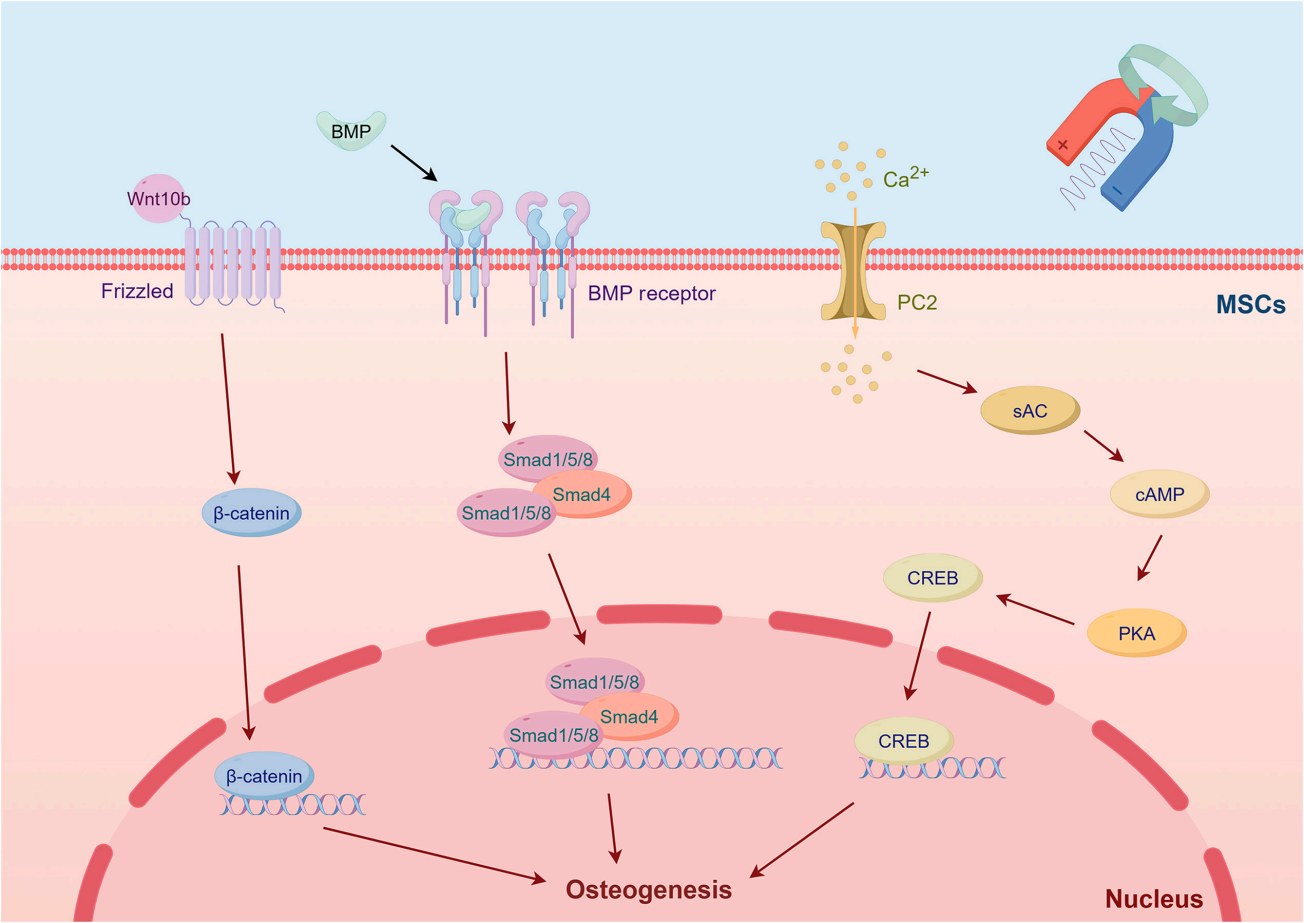
The mechanisms of PEMF-induced osteogenic differentiation of MSCs (by Figdraw). PEMFs cause Ca^2+^ influx in MSCs and stimulate them through pathways such as Wnt/β-catenin and BMP, thereby promoting their osteogenic differentiation.

Despite the diverse approaches of applying MFs in cellular and organismal research, a coherent clue emerges from the findings. Researches have shown that EMF stimulation activates cell surface adenosine A2A receptors, which, in turn, induces signaling through both canonical (Wnt/β-catenin-dependent) and noncanonical (Wnt/Ca^2+^-mediated) pathways of the Wnt signaling cascade [165]. Zhou et al. [166] have confirmed that sinusoidal EMFs up-regulate collagen-1α1 and Wnt10b in rats, driving osteogenic differentiation and maturation of rat calvarial osteoblasts through the Wnt10b/β-catenin pathway. They also clarified that PEMFs achieve the similar osteogenesis results by modulating intracellular cAMP levels and activating the sAC-cAMP-PKA-CREB signaling axis, leading to the phosphorylation and nuclear translocation of CREB [167]. Meanwhile, Li et al. [168] found out that sinusoidal EMF promotes osteogenic differentiation of BMSCs by up-regulating the gene expression of BMP receptors (BMPR1A, BMPR1B, and BMPR2) and associated signaling components (Smad4 and Smad1/5/8) (Fig. 4), as well as via the mitogen-activated protein kinase (MAPK), extracellular signal-regulated kinase (ERK), and c-Jun N-terminal kinase (JNK) pathways [27].

Further evidence continued to enhance the understanding of the biological functions of EMFs. Sinusoidal EMFs enhance FGF’s role in osteogenic differentiation of the periodontal ligament stem cell through the rapidly accelerated fibrosarcoma (RAF)/mitogen-activated protein kinase kinase (MEK)/ERK pathway [169]. PEMF has been shown to up-regulate Wnt ligands in type 2 diabetes mellitus (T2DM) mice, activating the Wnt/β-catenin pathway and improving bone quality and cellular bioactivity [29]. Meanwhile, dynamic rotating fields intervene in the receptor activator of nuclear factor-κB ligand (RANKL)/receptor activator of nuclear factor-κB (RANK)/osteoprotegerin (OPG) pathway in ankylosing spondylitis mice, maintaining bone homeostasis and immune balance [170]. Although a multitude of studies have delved into the mechanisms of EMFs, the majority of research has focused on moderate field ranging from 1 mT to 1 T. The potential effects of EMFs outside this intensity range remain largely unexplored.

Different from EMFs, the application of SMFs is more extensive, with mechanisms gradually being unveiled. Based on research into SMFs of varying intensities, the current academic consensus holds that hypomagnetic fields may lead to bone loss and are detrimental to bone regeneration. In contrast, strong MFs promote the differentiation of osteoblasts. Studies have indicated that a hypomagnetic field of <300 nT can trigger oxidative stress responses in rats. Excessive ROS stimulate osteoblasts to secrete receptor activator of the NF-κB ligand, which promotes the maturation and activation of osteoclasts, ultimately leading to bone resorption [42]. On the other hand, a high SMF of 16 T increases the generation of nitric oxide (NO), inhibiting the differentiation of osteoclasts and thereby reducing bone resorption [38]. As for a moderate SMF (0.2 to 0.6 T), it inhibits PPARγ-mediated gene expression, activates Runx2 signaling, and induces osteogenic differentiation in BMSCs [34,171].

These findings highlight the specificity of MF effects on cellular processes and the heterogeneity of intracellular responses to different magnetic stimuli. While the field is yet to be systematically mapped, emerging literature provides valuable insights and directions for future research.

### Effects of mechanical stimuli by magnetic materials under MFs

**Fig. 5.**
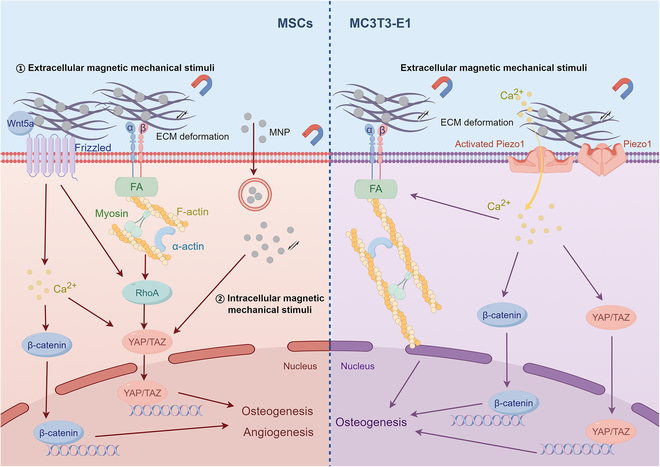
The mechanisms of magnetic mechanical stimulation on cells (by Figdraw). MNPs exert mechanical stimuli on BMSCs and preosteoblasts in response to an external MF, primarily through signaling pathways mediated by Wnt/β-catenin, integrin/RhoA/YAP, and Piezo1.

Magnetic mechanical stimulation exerts effects on a broad range of cell types, triggering a series of intracellular events that modulate cellular functions and behaviors (Fig. 5).

Weng et al. designed a magnetic collagen coating and uncovered that the coating, when subjected to an MF, provides mechanical stimulation to MSCs. This stimulation predominantly modulates the cytoskeletal development and motility of MSCs, thereby activating the mechanotransduction pathways. Engagement of the Yes-associated protein (YAP)/transcriptional co-activator with PDZ-binding motif (TAZ) signaling pathway has been shown to promote osteogenic differentiation [107]. Moreover, the variability in the direction of mechanical forces may induce differential osteogenic outcomes through the α5β1 integrin-dependent RhoA/ROCK signaling pathway [106]. Complementing these findings, He et al. [172] have demonstrated that MGO within an SMF facilitates the osteogenic differentiation of BMSCs via the Wnt/β-catenin pathway. In parallel, Yao et al. have delineated how the cooperative effect of MNPs and MFs facilitates bone formation, mineralization, and vascularization in magnetized MSCs. This process is mediated through the YAP/β-catenin signaling pathway [59], offering a nuanced understanding of the interplay between magnetic stimuli and cellular responses in the context of bone regeneration.

MNPs within an SMF have been shown to enhance osteogenic differentiation in MC3T3 cells by increasing the activity of RUNX-2, a master transcription factor in osteoblastogenesis. Additionally, MNPs induce apoptosis via mitochondrial-dependent pathways, which suppress osteoclast function, offering a potential strategy for modulating bone remodeling [173].

Within similar experimental paradigms, magnetic scaffolds or nanocomposite coatings effectively transduce mechanical stimuli to MC3T3 cells. This process is coupled with the up-regulation of Piezo1, a mechanosensitive ion channel [95,113], and the activation of integrin-mediated RhoA/ROCK and MEK/ERK signaling pathways [112,174,175], which, in turn, facilitates the osteogenic differentiation of these cells. These approaches explore the mechanisms of magnetic scaffolds to make use of mechanical cues in manipulating the fate of osteoblastic cells.

Moreover, these magnetic scaffolds further activate integrin and BMP-2 signaling pathways of osteoblasts in the presence of an SMF [176]. This multimodal approach takes the advantage of both the mechanical and magnetic properties of the scaffolds to regulate a favorable cellular response, thereby enhancing the therapeutic potential of bone tissue engineering strategies.

In addition to the discussed mechanisms, contemporary research suggests that magnetic mechanical stimulation is instrumental in the immunomodulatory processes that promote bone healing. Specifically, it is involved in guiding the differentiation of macrophages toward an M2-like phenotype, which is crucial for tissue repair and resolution of inflammation. This immunomodulation is mediated through a series of intracellular signaling events: the initiation of integrin-associated cascades, the attenuation of JNK phosphorylation in the MAPK pathway [106], the regulation of the Rho/ROCK signaling axis [85], the suppression of TLR2/4 receptor activation, and the potentiation of VEGFR2 signaling [87]. These coordinated actions exemplify the sophisticated interplay between magnetic mechanical stimulation and immune cell behavior in the context of bone regeneration.

### Effects of Fe^3+^ releasing of magnetic materials

The prevalence of iron in most magnetic biomaterials has directed research focus toward the role of Fe^3+^. Studies have indicated that in the presence of Fe^3+^ ions, there is an increased proliferation of BMSCs and HUVECs, which stimulates osteogenic and angiogenic processes [177]. Li et al. [178,179] have also reported that the inherent magnetic properties and the minute release of Fe^3+^ ions have been shown to enhance osteoblast proliferation, up-regulate the expression of osteogenic markers, increase collagen secretion, and promote the mineralization of the extracellular matrix. However, it should be noted that elevated Fe^3+^ concentrations have the potential to induce cytotoxicity, and iron overload may result in osteocytes apoptosis [177,180]. These findings highlight the nuanced influence of Fe^3+^ on magnetic-driven bone regeneration and the critical balance required to take advantage of its osteogenic potential without causing adverse cellular effects.

Current mechanistic studies have revealed that although our understanding of magnetism remains merely the tip of the iceberg, it is undeniable that both MFs themselves and the various magnetic effects generated by MNPs responding to external fields exert profound impacts on biological systems. Notably, the in situ implantation or targeted delivery of magnetic materials often leads to iron ion accumulation, encompassing not only Fe^3+^ but also ferrous Fe^2+^ ions, which are inextricably linked to cellular ferroptosis. While surface modification strategies have been employed to mitigate the toxicity of MNPs, it remains imperative to address their metabolic fate in vivo to avoid prolonged retention and subsequent adverse effects.

## MDRM in Clinical Applications

In recent years, MDRM has been beginning to demonstrate its potential in clinical treatment. With the advent of the concept of magnetic surgery, our team has independently developed a comprehensive system of 6 magnetic surgical technologies, including magnetic anastomosis, magnetic anchoring, magnetic navigation, magnetic levitation, magnetic tracing, and magnetic driving [181–186]. These technologies have been widely applied in various surgical procedures within the fields of hepatobiliary surgery, general surgery, and pediatric surgery for a range of diseases and have demonstrated remarkably positive results in both tissue reconstruction and the restoration of organ functionality. The application of magnetic-related technologies has simplified surgical procedures, reduced operation times, decreased operational difficulty, and enhanced the precision of treatment, bringing revolutionary changes to surgical operation and setting a new benchmark in the international medical field.

In the field of orthopedics, magnetic-related medical devices are also used in clinical treatment. Following the FDA approval of PEMF application in fracture healing therapy [187–189], numerous PEMF devices have been widely implemented in clinical practice, accompanied by comprehensive clinical investigations. Certain studies demonstrate that timely PEMF intervention, whether following closed reduction or surgical treatment of fractures, effectively enhances bone regeneration, elevates healing success rates, and reduces recovery duration [188,190–196]. This allows patients to achieve functional reconstruction earlier, such as opening their mouths after a mandibular fracture [191]. However, conflicting research outcomes have emerged, with one trial indicating no statistically significant acceleration of healing timelines through PEMF application [197]. Therefore, while the therapeutic efficacy of PEMF has been validated, the optimization of treatment parameters—particularly concerning acute fractures, delayed unions, and nonunions—continues to warrant rigorous exploration to establish standardized clinical guidelines.

Magnetic effect-based medical devices utilize magnetic forces as the core therapeutic modality in orthopedic clinical practice, achieving deformity correction or distraction osteogenesis through mechanical stimulation. Magnetically controlled growing rods for scoliosis correction employ noncontact magnetic force transmission, enabling external adjustment of the implanted device’s length via a controller [198–201]. This method facilitates spinal correction and optimizes the restoration of spinal function. Meanwhile, magnetically controlled intramedullary limb lengthening system function is based on analogous principles. Their utilization can substantially extend shortened limbs in patients due to their magnetic-controlled bone transport mechanisms, which facilitate the regeneration and functional reconstruction of bone tissue [202–205]. Additionally, the development of the magnetic mandibular distractor exemplifies the practical application of magnetic force based on the theory of distraction osteogenesis [206,207]. These medical devices facilitate bone regeneration and tissue repair by employing magnetic force, thereby contributing to the restoration of the body’s original functions. Compared to conventional therapeutic approaches, they offer the advantages of minimal invasiveness, lower infection rates, and more convenient regulation.

The current application of MDRM in orthopedic disease management remains primarily focused on 2 clinical objectives: facilitating fracture healing or addressing nonunion bone defects, and implementing functional reconstruction through magnetic force-mediated deformity correction. Therapeutic interventions targeting cases with concurrent infections or multifactorial pathological complexity remain confined to experimental exploration in laboratory settings. While emerging evidence suggests the potential immunomodulatory and anti-inflammatory properties of MFs [208–210], prudent clinical judgment must be exercised when considering magnetotherapy for complex orthopedic conditions. This caution stems from the microcirculation and hemodynamic enhancement induced by MFs [211,212], which may paradoxically contribute to localized infection dissemination and other adverse outcomes in specific clinical scenarios. These critical safety considerations necessitate further investigation through rigorously designed multicenter trials. Only upon acquisition of robust, high-level clinical evidence can definitive guidelines be established to ensure the safe and effective implementation of magnetic therapies in managing intricate orthopedic pathologies.

## Conclusion and Future Prospect

Researches on MDRM for bone repair have made significant progress in the synthesis and application of materials, with magnetic force effects achieving multidimensional and multifaceted stimulation in particular. However, during the application of magnets, there are numerous confounding factors, a wide range of dimensions involved, and complex scenarios, which pose challenges to unraveling the mechanisms involved. It is difficult to comprehensively understand the mechanisms of how magnets affect cells and tissues across multiple systems. Additionally, the limitations of observation methods present another major challenge in accurately evaluating the outcomes of magnetic applications. It is crucial for researchers to overcome scientific obstacles, such as controlling the variables of magnetic effects, improving the logical framework of magnetic actions, and quantifying the results of magnetic interventions.

To further drive the clinical translation of magnetic materials, it is essential to explore their metabolic fate within the human body. Although some researchers have made preliminary explorations into the life cycle of MNPs in MSCs and elucidated their functions in different differentiation states [213], or evaluated the distribution and metabolism of MNPs in mice [214], this only scratches the surface in understanding the vast array of magnetic materials. Furthermore, the investigation into the biocompatibility of magnetic materials should not be confined merely to the cellular level. In the future, we can further explore their complex metabolic pathways and toxicity at the organ or even systemic level through explant culture model studies or the construction of organoids. Concurrently, given the extensive applications of MFs, an assessment of their safety cannot be overlooked either. Although current studies have conducted preliminary explorations using strong, medium, and weak SMFs, achieving notable results, research on MFs still warrants further refinement to deepen our understanding and facilitate their translation to clinical therapy.

Nowadays, although researchers have developed several products for clinical applications based on the existing understanding of magnets, the translation potential of magnetic materials and magnetic technologies remains vast. The applications of magnet-controlled growing rods and the magnet-controlled intramedullary limb lengthening system have achieved significant success, yet the potential of MDRM in bone regeneration can be further expanded. The biomechanical stimulation mediated by the deformation of magnetic responsive materials under MFs holds a pivotal role in bone reconstruction [215], suggesting a promising future for the development of magnetic surgical instruments and implants. However, the current magnetic implants also have certain limitations. For instance, post-implantation restrictions on magnetic resonance imaging (MRI) and the distortion of soft tissues impede the precise transmission of externally applied magnetic interventions into the body. Based on the limitations, Bian et al. [216] investigated MRI safety and biocompatibility of the iron bioresorbable scaffold for the first time and proposed a systematic methodology for MRI evaluation of iron bioresorbable scaffolds. In future research, we may broaden their application range and enhance their therapeutic efficiency by optimizing material composition and adjusting size and structure. Meanwhile, investigating the iron metabolism mechanisms of MNPs in vivo and reducing their accumulation risks have become unavoidable challenges in MDRM. In research focused on utilizing MFs as standalone noninvasive therapeutic approaches, it is imperative to establish an MF parameter database through big data and AI-driven analysis and screening, complemented by multicenter clinical studies. This should be conducted under the premise of safety and scientific rigor, while simultaneously developing specialized therapeutic equipment and advancing standardized treatment guidelines.

In summary, despite the extraordinary achievements MDRM has accomplished in the structural regeneration and functional restoration of bone, most advancements are still in the purview of laboratory investigations. This is largely due to the inadequate interdisciplinary integration and insufficient understanding of MDRM. However, advancements in artificial intelligence, advanced manufacturing technologies, single-cell sequencing, multi-omics, and spatial transcriptomics are poised to elucidate the underlying mechanisms of MDRM. These developments will lead to the creation of safer and more efficient strategies and devices for MDRM, thereby facilitating its translation to clinical applications.
